# Supplementary figures and images for: Impact Assessment of Free-Roaming Dog Population Management by CNVR in Greater Bangkok
Source: Animals (Basel). 2023 May 23;13(11):1726. doi: 10.3390/ani13111726 (PMC10252118; doi:10.3390/ani13111726)

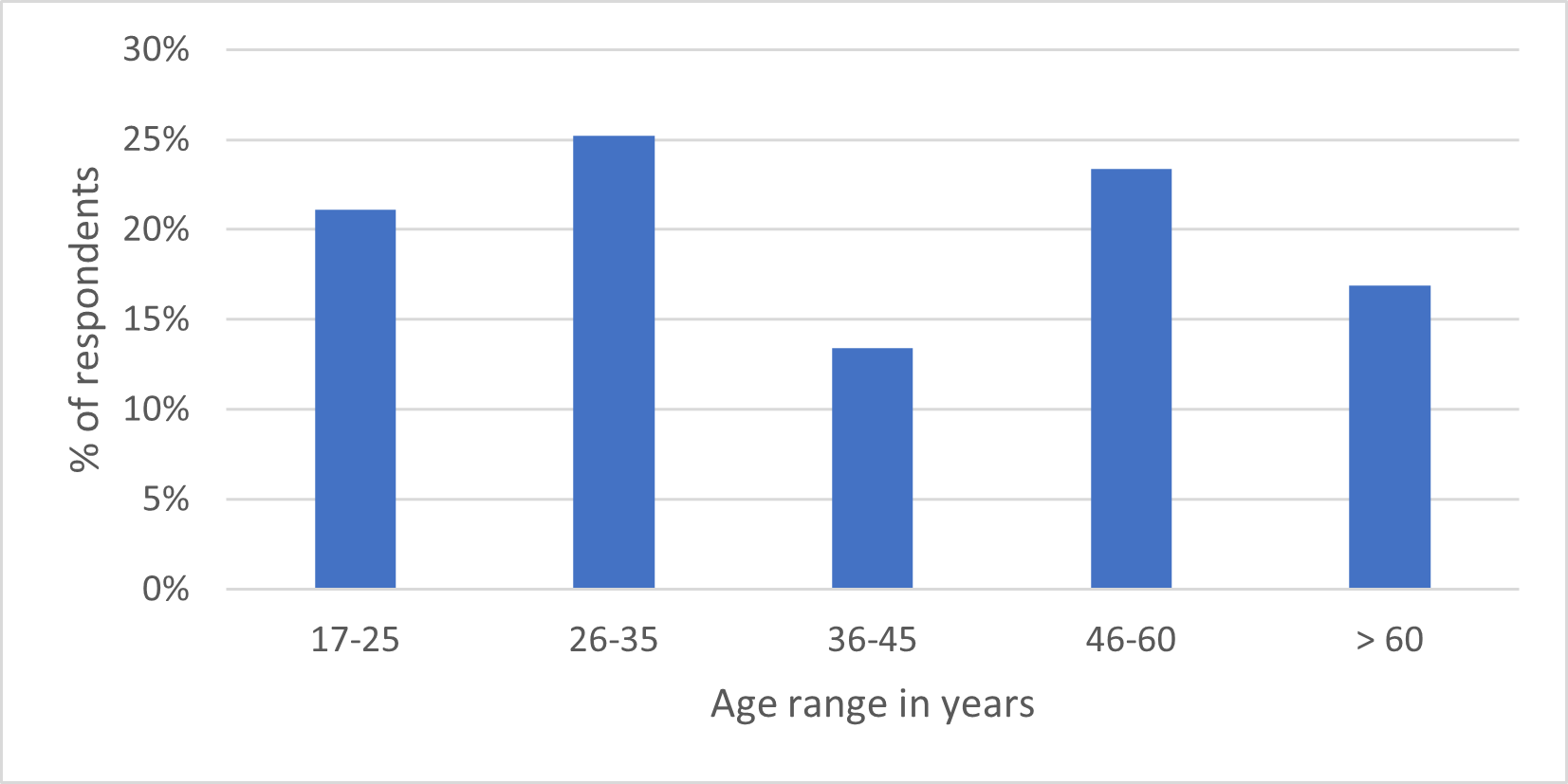

Supplement: Supplementary file 1 [file animals-13-01726-s001.zip › S4 - Age distribution.png]

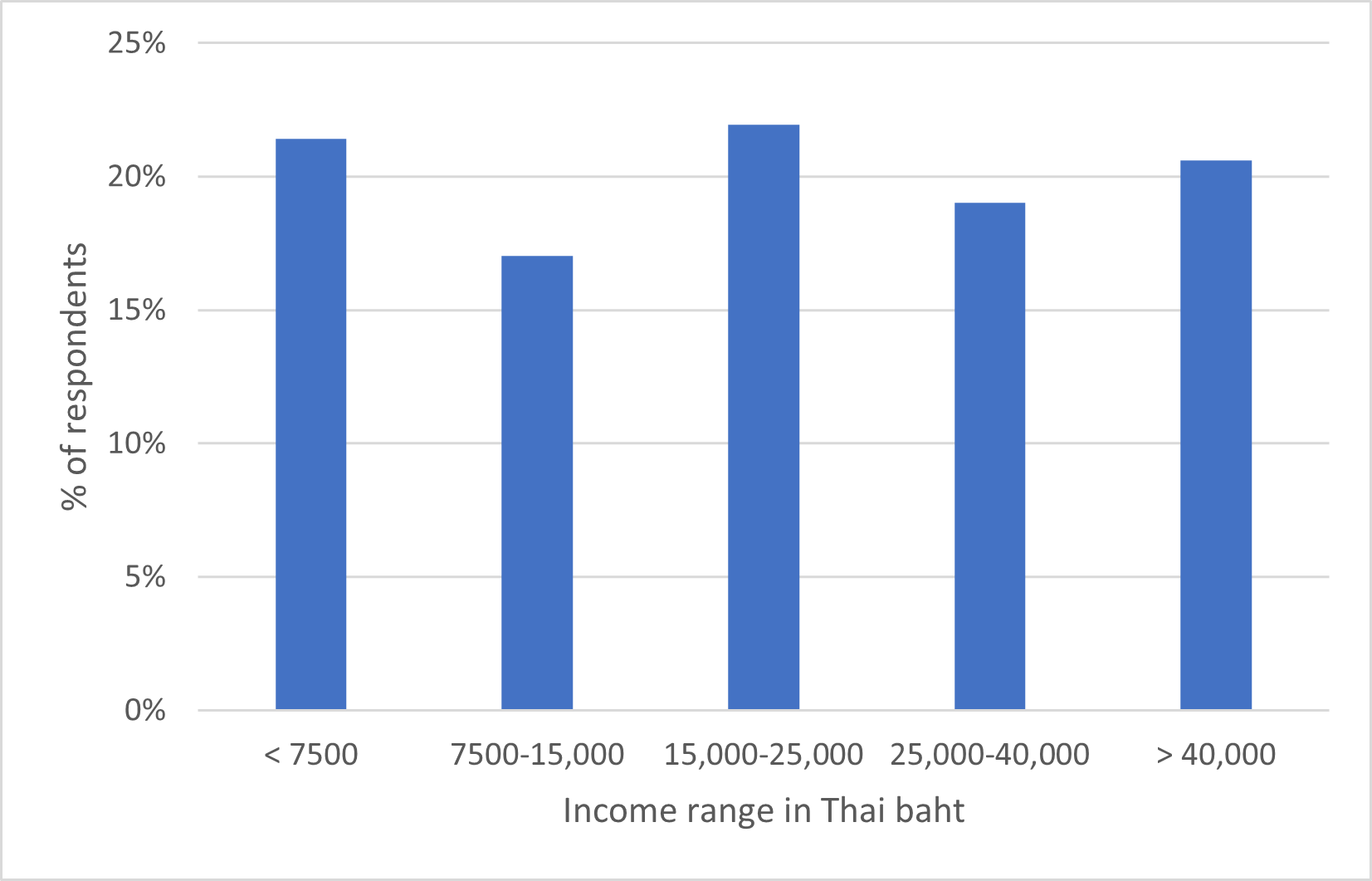

Supplement: Supplementary file 1 [file animals-13-01726-s001.zip › S5 - Income distribution updated 19 May.png]
