# Supplementary material for: Impact Assessment of Free-Roaming Dog Population Management by CNVR in Greater Bangkok
Source: Animals (Basel). 2023 May 23;13(11):1726. doi: 10.3390/ani13111726 (PMC10252118; doi:10.3390/ani13111726)
Supplement: Supplementary file 1 [file animals-13-01726-s001.zip › S1 - SDF SOPs for CNVR.zip!/S1 - SDF SOPs for CNVR.pdf]

www.soidog.org

| | | |
|---|---|---|
| **Number** | **:** | **CNVR/SOP001** |
| **Division** | **:** | **CNVR Thailand** |
| **Date Written** | **:** | **29th November 2022** |
| **Last Changed** | **:** | **-** |
| **Written By** | **:** | **Tuntikorn Rungpatana, DVM** |
| **Title/ Subject** | **:** | **Operation time and weekend** |

---

Purpose : To specify working hours for Mobile Unit staff.

1. Bangkok Mobile Teams: work Monday to Friday (5 day week) from 8:00 AM to 5:00 PM. Weekends off.

2. Southern Mobile Teams: work Monday to Saturday (6 day week) from 8:00 AM to 5:00 PM for 3 weeks in a row, then 5 days off.

    - AROs (dog catchers) will make an effort to locate dogs and bring them to the Mobile Unit by 8 AM and no later than 3:30 PM.

    - After the predetermined number of dogs have been sterilised and the last dog has recovered, the dogs will be loaded onto the truck and taken back to their territories. After all dogs have been loaded onto truck and the Mobile Unit deep cleaned, staff may leave for the day.

---

www.soidog.org

| | | |
|---|---|---|
| **Number** | **:** | **CNVR/SOP002** |
| **Division** | **:** | **CNVR Thailand** |
| **Date Written** | **:** | **29th November 2022** |
| **Last Changed** | **:** | **-** |
| **Written By** | **:** | **Tuntikorn Rungpatana, DVM** |
| **Title/ Subject** | **:** | **Dog types - Classification** |

---

Purpose : To clarify differences between types of free-roaming dogs encountered by AROs & at the Mobile Unit (codes used in data collection, interpretation, etc).

- "S" refers to a Stray or feral dog. This is a dog that has reverted to the wild state and is not dependent on humans for survival.
- "C" refers to a Community dog. This type of dog has at least one feeder. These dogs live in communities such as markets, temples, schools, in front of convenience stores, etc and interact with people.
- "O" refers to a free-roaming owned dog. These dogs have an owner, but are allowed to roam freely for all of or part of the day.
- "O**" refers to an owned dog which is always kept in a fenced-in location (ie NOT free-roaming).

Note: Soi Dog's CNVR Programme sterilises all dog categories, however free-roaming dogs are done as a priority. O** category should only be sterilised at Mobile Unit if appropriate or urgent (eg poor owner, pyometra or pregnant dog & local vet not willing to abort).

---

www.soidog.org

| | | |
|---|---|---|
| **Number** | **:** | **CNVR/SOP003** |
| **Division** | **:** | **CNVR Thailand** |
| **Date Written** | **:** | **29th November 2022** |
| **Last Changed** | **:** | **-** |
| **Written By** | **:** | **Tuntikorn Rungpatana, DVM** |
| **Title/ Subject** | **:** | **Animal Criteria** |

---

Purpose : To quantify dogs' and cats' minimum age for sterilisation and vaccination in CNVR programme.

The approximate age for safe sterilisation of a dog is 10 weeks old, and 12 weeks old for a cat, either male or female AS LONG AS:

- Dog >> All 28 milk teeth grow and weight is minimum 2 kilograms.
- Cat >> All 26 milk teeth grow and weight is mimimum 1 kilogram.

All animals going through CNVR should be vaccinated combo **+ rabies** (including puppies and kittens. If they are old enough to be sterilised safely, they are old enough to receive a rabies vaccine too)

---

| | | |
|---|---|---|
| **Number** | **:** | **CNVR/SOP004** |
| **Division** | **:** | **CNVR Thailand** |
| **Date Written** | **:** | **29th November 2022** |
| **Last Changed** | **:** | **-** |
| **Written By** | **:** | **Tuntikorn Rungpatana, DVM** |
| **Title/ Subject** | **:** | **Working area and Animal Intake** |

---

Purpose : To limit the working area for Mobile Units in order to achieve an intensive effort and high % coverage quickly.

1. AROs to collect only dogs that live in the current working area for Mobile Team ie in an area that had been surveyed pre-intervention, and only with permission from HOD.
2. If border area of Greater Bangkok ie areas adjacent to other provinces such as Ratchaburi, Samut Songkhram, etc, AROs can bring dogs from within 2km of provincial boundary where Mobile Unit is stationed.
3. Do not capture dogs in the next area the Team plans to enter, without authorisation from HOD.
4. Do not schedule appointments to pick up dogs for sterilisation from outside the Mobile Unit's area or from areas that have never been surveyed.
5. When dealing with walk-ins and requests to sterilise owned dogs, request the owner's address precisely (this can be found on the owner's identification card).
6. If an owned dog comes from outside the current working area (such as the same province, but a different district), the vet must consider whether the case is appropriate and/ or urgent.
    a. If the dog is pregnant, has pyometra or another emergency situation then we will sterilise.
    b. In non-emergency situations, decline and inform the dog's owner to wait until the Mobile Unit moves to their area or to use a private vet clinic.

7. Freelance dog catchers must inform the Logistics Manager and gain approval, before bringing dogs into the Mobile Unit.
8. Do not sterilise the dogs brought in by freelance dog catchers without authorisation from HOD.
9. Do not allow SDF's dog catcher to arrange to transfer dogs from a freelance dog catcher on the way to the Mobile Unit.

---

| | | |
|---|---|---|
| Number | : | CNVR/SOP005 |
| Division | : | CNVR Thailand |
| Date Written | : | 29th November 2022 |
| Last Changed | : | - |
| Written By | : | Tuntikorn Rungpatana, DVM |
| Title/ Subject | : | General medicine and anaesthesia |

---

Purpose: To maximise the standard of general medicine and animal anaesthesia at SDF Mobile Units and avoid malpractice.

1. Only the veterinarian can make the decision on the medical problems; they must follow SDF protocols with animal welfare as a priority and not let feeders or other members of the public influence their decisions.
2. The veterinarians have to inspect all animals brought in for overall body condition, demeanor, any difficulties breathing, pale mucous membrane and sign of upper respiratory disease including nasal discharge. Veterinarians have to do a full general health check on animals that are suspected of having the health problems before premedication is given. If the dog is not fit for surgery, it should not be operated on.
3. If other health problems are found during pre-op, intra-op, or during post-op, the dog needs to be evaluated and treated by the veterinarian.
4. Before starting surgeries for the day a CPR area must be prepared, ready with oxygen, crash-box, etc.
5. The anaesthetic machines must be checked before surgery starts every day by vet technicians or vet nurses.
6. Only 1 animal can be maintained on 1 anaesthetic machine.
7. Veterinary support staff must be kept on hand to monitor the animal during pre-op, intra-op, and during post-op period, to make sure the dog is kept under adequate anaesthesia and recovers smoothly.

8. The anaesthetic drugs must be used under the veterinarian's control.
9. Veterinary support staff are responsible for pre-med, induction, and preparing animals under the supervision of a veterinarian and in accordance with the SDF protocol (drug, dosage, and administration methods), and maintain aseptic techniques in the process of cleaning equipment, daily unit cleaning, weekly unit cleaning and deep cleaning the unit when infectious disease is found.
10. All veterinarians, vet technicians, and vet nurses working at a Mobile Unit must be able to do cardiopulmonary resuscitation (CPR).

| | | |
|---|---|---|
| **Number** | **:** | **CNVR/SOP006** |
| **Division** | **:** | **CNVR Thailand** |
| **Date Written** | **:** | **29th November 2022** |
| **Last Changed** | **:** | **-** |
| **Written By** | **:** | **Tuntikorn Rungpatana, DVM** |
| **Title/ Subject** | **:** | **Aseptic technique** |

---

Purpose : To maximise the standard of hygiene in mobile clinic and avoid surgical contamination.

All of the CNVR staff must strictly follow the aseptic technique procedure under the control of the veterinarian

- The surgical instrument steam sterilisation follows the CDC Guideline for Disinfection and Sterilization in Healthcare Facilities, 2008(page 58-60).
- Single use of surgical equipment ONLY.
- Vet support staff is responsible for cleaning and preparation of surgical sets, which must be ready and in sufficient quantity (40 M and 40 F sets) for the day ahead.
- The surgical blade, green drape, window drape, and surgical gloves must be single-use and changed every time a new animal is operated on.
- The preparation process (pre-op) must be followed by SDF Vet Support Staff Guidelines.

---

| | | |
|---|---|---|
| **Number** | **:** | **CNVR/SOP007** |
| **Division** | **:** | **CNVR Thailand** |
| **Date Written** | **:** | **29th November 2022** |
| **Last Changed** | **:** | **-** |
| **Written By** | **:** | **Tuntikorn Rungpatana, DVM** |
| **Title/ Subject** | **:** | **Cleaning policies** |

---

Purpose : To standardise cleaning and sanitation procedures at SDF Mobile Units

1. **Daily cleaning**

    1. For sterilisation area

        - Clean area with general detergent and use mop & bucket or scrubbing brush to remove soiling and organic debris.

        - Completely rinse with running water all detergent residues and squeegee excess water from floor and allow to dry.

    2. Cages and Trucks

        - Remove soiling and organic debris.

        - Clean cages and truck with Chlorhexidine scrub or proper vehicle soap (detergent).

        - Completely rinse all the detergent off with running water, absorb excess water by towel and allow to dry.

2. **Weekly cleaning (on last day)**

    1. For sterilisation area

- Clean area with general detergent and use mop & bucket or scrubbing brush to remove soiling and organic debris.
- Completely rinse with running water all detergent residues and squeegee excess water from floor and leave to dry.
- Disinfect area by Bleach (Haiter 6%) prepared 1:20 – 1:30 dilution, 40 ml bleach dilute in 1 litre of water, leave wet for minimum 15 mins then rinse thoroughly and allow to dry.

2. Cages and Trucks
   - Remove soiling and organic debris.
   - Clean cages and truck with Chlorhexidine scrub or proper vehicle soap (detergent).
   - Completely rinse all the detergent off with running water, absorb excess water by towel and allow to dry.
   - Disinfect cage and truck trays by Bleach (Haiter 6%) prepared 1:20 – 1:30 dilution, 40 ml bleach dilute in 1 litre of water, leave wet for minimum 15 mins then rinse thoroughly and absorb excess water by towel and allow to dry.
3. For equipment or materials that cannot rinse by water use only Alcohol 70% spray after cleaned, and leave to dry.

**3. Contaminated (in-contact with CDV/ CPV/ FPV)**

1. For sterilisation area
   - Clean area with general detergent and use mop & bucket or scrubbing brush to remove soiling and organic debris.

www.soidog.org

- Completely rinse with running water all detergent residues and squeegee excess water from floor and allow to dry.
- Disinfect area by Virkon prepared 1:100 measured by WEIGHT,
    - For cleaning use 175 ml of Virkon powder = 200g., dilute in 20 litres of water.
    - For spray use ¼ of scoop cup or 4.5 ml of Virkon powder = 5g., dilute in 500 ml of water in spray bottle.

    Leave wet for maximum 10 mins then rinse thoroughly and allow to dry.

2. Cages and Trucks

- Remove soiling and organic debris.
- Clean cages and truck with Chlorhexidine scrub or proper vehicle soap (detergent).
- Completely rinse all the detergent off with running water, absorb excess water by towel and allow to dry.
- Disinfect cage and truck trays by Virkon prepared 1:100 measured by WEIGHT,
    - For cleaning use 175 ml of Virkon powder = 200g., dilute in 20 litres of water.
    - For spray use ¼ of scoop cup or 4.5 ml of Virkon powder = 5g., dilute in 500 ml of water in spray bottle.

    Leave wet for maximum 10 mins then rinse thoroughly and absorb excess water by towel and allow to dry.

3. For equipment or materials that cannot rinse by water use only Alcohol 70% spray after cleaned, and leave to dry.

Note:

- Virkon is an oxidising agent - it will accelerate the rusting of metals and damage the paint. 10 minutes contact time is the manufacturers maximum recommended contact time.
- Both Virkon and bleach should be used within a short period of time after being diluted with water.
    - Virkon is stable for up to 72hrs, if Virkon is no longer pink, it is NOT active - the pink colour is a pH indicator that tells us if the Virkon is strong enough to work. If it is weak pink or clear, discard and make up new.
    - bleach should be used within 24hrs - it is not stable and will degrade to NaCl and water in a very short period of time
- 70% alcohol will not destroy most viruses. For delicate implements, some laboratories will use a 1:200 Virkon solution and wipe the instruments over, then use 70% alcohol to remove the Virkon. CPV and CDV are not killed by alcohol. Any implement that can safely be immersed in Virkon, should be.
- Treated surfaces must remain WET with disinfectant for 10 minutes - this may mean you need to reapply
    - spray and wipe off is not enough
    - fine spray with a spray bottle is not enough
    - Surfaces must be dripping wet for 10 minutes

CNVRSOP008.1-Cage's card

| | | | |
|---|---|---|---|
| **No.** | | **DC** | |
| **F** | | **M** | |
| **Weight** | | | |
| **S** | **C** | **O** | **O*** |
| **1** **(DC)** | **2** **(Net)** | **3** **(FD,O)** | **4** **(Dart)** |
| **Dart** | **Z** | **X** | |
| **Location** | | | |
| **Name** | | | |
| **Tel.** | | | |
| **Vaccine** | | **IVM** | |
| **Note** | | | |

| | | |
|---|---|---|
| **Number** | **:** | **CNVR/SOP008** |
| **Division** | **:** | **CNVR Thailand** |
| **Date Written** | **:** | **29th November 2022** |
| **Last Changed** | **:** | **-** |
| **Written By** | **:** | **Tuntikorn Rungpatana, DVM** |
| **Title/ Subject** | **:** | **Animal resting and handling** |

---

Purpose: To maximise animals' comfort and wellbeing while at Mobile Unit.

1. The maximum number of dogs:

   1 adult dog per cage

   2 puppies per cage

2. Place plastic base (bottom of travel crate) in each cage and always keep it clean.
3. If a TVT dog is discovered, keep it in a cage and away from the sterilisation area.
4. AROs must weigh dog before placing in the cage.
5. AROs must write down dogs' details on the information card outside the cage, including darting drugs, previous health issues, feeder's tel no and etc.. Also add in consent form from owner, if applicable.
6. The puppies must be transported in a cage or separated from adults while travelling in the truck.
7. Always handle animals in a gentle way, at the same time taking care not to get bitten. Use muzzles on adult dogs when moving from truck to cage and from cage to truck.

8. The dogs must fully recover from anaesthetic before being loaded onto truck, to take them back to their territories.

9. Follow the information of cage's card in **CNVR/SOP008.1**

---

www.soidog.org

| | | |
|---|---|---|
| **Number** | **:** | **CNVR/SOP009** |
| **Division** | **:** | **CNVR Thailand** |
| **Date Written** | **:** | **29th November 2022** |
| **Last Changed** | **:** | **-** |
| **Written By** | **:** | **Tuntikorn Rungpatana, DVM** |
| **Title/ Subject** | **:** | **Communication with owner/ feeder** |

---

Purpose: To improve the effectiveness of communication with feeders and owners in order to achieve animal health information which could affect the CNVR process.

1. If the dog is owned, **the owner** must accept the risk of anaesthetic and sign the consent form before the dog is taken to Mobile Unit.
2. Inform the owner/ feeder about the side-effects of anaesthesia drugs, risks involved (due to anaesthetic or possible underlying health issues that are non-detectable by physical exam only).
3. Write phone number of dog catcher or Mobile Unit phone before handing out SDF brochure.
4. Collect medical history, from owner or feeder, including last vaccination and ivermectin injection, then record information on the card in front of the cage and in the patient record.
5. All Mobile staff should be able to communicate clearly regarding pre and post op care as well as prophylaxis. Only the veterinarian is allowed to speak to the owner/ feeder regarding medical issues (eg pre/ intra/ post op complications, and other) & must call owner/ feeder in case of death.
6. Must inform the owner/ feeder (person responsible for the dog) before returning it.
7. Communicate with the dog's owner/feeder with respect, tenderness, and a service mind.

---

www.soidog.org

| | | |
|---|---|---|
| **Number** | **:** | **CNVR/SOP010** |
| **Division** | **:** | **CNVR Thailand** |
| **Date Written** | **:** | **29th November 2022** |
| **Last Changed** | **:** | **-** |
| **Written By** | **:** | **Tuntikorn Rungpatana, DVM** |
| **Title/ Subject** | **:** | **Driving Policy** |

---

Purpose: To minimise the problems for AROs transporting the dogs to Mobile Unit and optimise the standard of animal transportation.

1. Drive carefully and be responsible for others, people and animals.
2. Don’t drive over 100km/hr; in build-up residential areas not over 20km/hr.
3. Do not drive out of the routes unless instructed by HOD.
4. When an accident occurs, follow the protocol in the truck.
5. Animal rescue officers are responsible for repair and maintenance checks on their trucks, as well as daily and weekly cleaning of their trucks.
6. When the truck has broken down or needs to be repaired, AROs must inform HOD for permission to proceed.

---

www.soidog.org

| | | |
|---|---|---|
| Number | : | CNVR/SOP011 |
| Division | : | CNVR Thailand |
| Date Written | : | 29th November 2022 |
| Last Changed | : | - |
| Written By | : | Tuntikorn Rungpatana, DVM |
| Title/ Subject | : | Ear Tattoo and Code |

---

- All free roaming dogs and cats sterilised by the SDF Mobile Units must have an ID in the form of an ear tattoo.
- Cat ear tattoos – only two digits, the first digit represents the year that cat was sterilised, and the second digit represents the province.
- Dog ear tattoos - 3 digits that represent the year, month, and province, as below:

| 1st CODE | Year | 2nd CODE | Month | 3rd CODE | Province |
|---|---|---|---|---|---|
| M | 2013 | A | January | A | Bangkok |
| N | 2014 | B | February | B | Pathum Thani |
| O | 2015 | C | March | C | Samut Sakhon |
| P | 2016 | D | April | D | Nakhon Pathom |
| Q | 2017 | E | May | E | Samut Prakan |
| R | 2018 | F | June | F | Nonthaburi |
| S | 2019 | G | July | G | |
| T | 2020 | H | August | H | |
| U | 2021 | I | September | I | |
| V | 2022 | J | October | J | |
| W | 2023 | K | November | K | |
| X | 2024 | L | December | L | |
| Y | 2025 | | | M | |
| Z | 2026 | | | N | |
| A | 2027 | | | O | Narathiwat |
| B | 2028 | | | P | Phuket |
| C | 2029 | | | Q | Yala |
| D | 2030 | | | R | Pattani |
| E | 2031 | | | S | Songkhla |
| F | 2032 | | | T | Satun |
| G | 2033 | | | U | Trang |
| H | 2034 | | | V | Krabi |
| I | 2035 | | | W | Phatthalung |
| J | 2036 | | | X | Surat Thani |
| K | 2037 | | | Y | Phangnga |
| L | 2038 | | | Z | Nakhon Si Thammarat |

| | | |
|---|---|---|
| **Number** | **:** | **CNVR/SOP012** |
| **Division** | **:** | **CNVR Thailand** |
| **Date Written** | **:** | **29th November 2022** |
| **Last Changed** | **:** | **-** |
| **Written By** | **:** | **Tuntikorn Rungpatana, DVM** |
| **Title/ Subject** | **:** | **Pre and Post clinic survey Protocol** |

---

## Pre-clinic survey

The pre-clinic survey provides a good opportunity to not only establish the number of roaming dogs living in the area covered by the mobile clinics, but also to collect enough information to facilitate later catching of the dogs. Hence, the pre-clinic protocol evolved:

- Using My Tracks on your phone, open the polygon that indicates the boundary of the Khet/sub-district. This indicates the extent of the catching area for the clinic and therefore the boundary of the survey.
- Drive along every Soi within the boundary, counting every roaming dog. Stop to investigate each dog, establishing its sterilisation status and asking local people whether there are feeders or owners associated with the dog and where other roaming dogs may be found.
- Record the total dogs for each Soi using 'Add a new point' in My Tracks using the following 4 digits code name:
    1. *Sterilisation*
        - *S = Sterilised*
        - *NS = Non-Sterilised*
        - *UN = Unknown*
    2. *Categories*
        - O = Owner (either confined or not)
        - C = Community dog (include feeder)
        - S = Stray or feral dog

3. Sex and Number
    - M…. = Male and Number
    - F…. = Female and Number
    - Fp…. = Female pregnant and Number
    - P…. = Puppies and Number
    - …. = only number meant unknown sex
4. Person contact
    - Name and tel. number

Example: **NS C F3 M2** means on this Soi there are 3 females and 2 males all unsterilised community dogs.

- My Tracks app. can export and share kml. file to email and then CNVR Admin can import them to My Map on web to summarise and generate data on the map.

**Post-clinic survey**

- Plan a route that takes you to known 'hotspots' where you are likely to see a lot of roaming dogs in a short space of time. Ensure this route achieves good coverage of the Khet/sub-district and is not restricted to just one part of the area, to include a representative sample of the roaming dog population in the survey.
- Record every dog you see on this route, not only the dogs observed at the hotspots.
- Record a minimum of 60 dogs on your post-clinic survey, more is always better.
    - With a sample of 60 dogs you can be 95% certain that the true coverage is within +/- 10% of your estimated coverage (e.g. you record the sterilisation status of 60 dogs and find 80% are sterilised, you can be 95% certain that between 70 and 90% of the wider dog population in that area is sterilised).
- Determine the overall sterilisation coverage percentage.  Divide the number of sterilised dogs observed by the total number of observed dogs.
- If the result of the Post-clinic survey is over 80 percent of number of observed dogs, then team can plan to move forward to next adjacent area.

| | | |
|---|---|---|
| **Number** | **:** | **CNVR/SOP013** |
| **Division** | **:** | **CNVR Thailand** |
| **Date Written** | **:** | **29th November 2022** |
| **Last Changed** | **:** | **-** |
| **Written By** | **:** | **Tuntikorn Rungpatana, DVM** |
| **Title/ Subject** | **:** | **Data collection & Reports** |

---

Purpose : To clarify what data we collect during CNVR process, as well as clarify reports generated.

**CNVR /SOP013.1 CNVR Daily report**

1. Veterinary support staff are responsible for summarising the total number of neutered animals by sex, age, category, and location in the patient's record. All CNVR Teams send patient records and summaries to CNVR Admin at the end of the day.

2. AROs must record every animal caught and drop a pin or marker on the online map.

3. If the dog or cat was brought in by owner or feeder, vet support staff must drop a pin or marker on the online map (Mobile Unit location).

4. Patient records and the marker in the online map must be cross-checked by the CNVR Admin to make sure they match, to ensure precision of the data.

**CNVR /SOP013.2 CNVR Weekly report**

1. The Logistics Manager must inform CNVR Admin before Thursday if Teams have a plan to change the location of Mobile Unit.

2. AROs must arrange for for pre and post clinic surveys during the week and send the file record (kml. file) to CNVR Admin to summarise, not over the weekend.

3. CNVR Admin collects and summarises the weekly report that is submitted to the HOD and the CNVR Director.

**CNVR /SOP013.3 CNVR Monthly report**

1. Mobile Veterinary Manager must summarise the monthly Team analysis and complications report and submit it to the CNVR Director.

2. CNVR Admin compile all CNVR information and submit a monthly report to the HOD and CNVR Director.

    a. In the timeline table, CNVR Admin will record the date of entry for the pre- and post surveys, including the date of entering the catching area and the date of exiting the area.

    b. Daily and Monthly Excel data tables contain the daily data on sterilisation from each region's Mobile Units by patient record and online map.

## Drug dosage for darting

| | | | | |
|---|---|---|---|---|
| Xylazine | 1 ml per 30 kg | **(Maximun 0.6 ml/dog)** | } | **IM ONLY** |
| Atropine | 1 ml per 40 kg | | | |
| Zoletil 100 | 1 ml per 20 kg | **(Maximum 1 ml/dog)** | | |

| Weight | Zoletil (IM) | Xylazine (IM) | Atropine (IM) |
|---|---|---|---|
| 5 | 0.25 | 0.16 | 0.125 |
| **6** | **0.3** | **0.2** | **0.15** |
| 7 | 0.35 | 0.23 | 0.175 |
| **8** | **0.4** | **0.26** | **0.2** |
| 9 | 0.45 | 0.3 | 0.225 |
| **10** | **0.5** | **0.33** | **0.25** |
| 11 | 0.55 | 0.36 | 0.275 |
| **12** | **0.6** | **0.4** | **0.3** |
| 13 | 0.65 | 0.43 | 0.325 |
| **14** | **0.7** | **0.46** | **0.35** |
| 15 | 0.75 | 0.5 | 0.375 |
| **16** | **0.8** | **0.53** | **0.4** |
| 17 | 0.85 | 0.56 | 0.425 |
| **18** | **0.9** | **0.6** | **0.45** |
| 19 | 0.95 | 0.6 | 0.475 |
| **20** | **1** | **0.6** | **0.5** |
| 21 | 1 | 0.6 | 0.525 |
| **22** | **1** | **0.6** | **0.55** |
| 23 | 1 | 0.6 | 0.575 |
| **24** | **1** | **0.6** | **0.6** |
| 25 | 1 | 0.6 | 0.625 |
| **26** | **1** | **0.6** | **0.65** |
| 27 | 1 | 0.6 | 0.675 |
| **28** | **1** | **0.6** | **0.7** |
| 29 | 1 | 0.6 | 0.725 |
| **30** | **1** | **0.6** | **0.75** |

| | | |
|---|---|---|
| **Number** | **:** | **CNVR/SOP014** |
| **Division** | **:** | **CNVR Thailand** |
| **Date Written** | **:** | **29th November 2022** |
| **Last Changed** | **:** | **-** |
| **Written By** | **:** | **Tuntikorn Rungpatana, DVM** |
| **Title/ Subject** | **:** | **Catching Policies** |

---

Purpose : To optimise the standard of catching methods and materials in both dogs and cats, for safe of both catcher and animal.

**Key points:**

- Humane catching and handling ONLY.
- Reduces stress in both animal and catcher.
- Minimises animal & human injuries.

Approaching :

- Approach dog slowly.
- Lower your body profile when approaching an animal.
- Avoid direct eye contact.
- Keep equipment at your side or behind your back.
- Minimise talking.
- Don't focus on catching the dog when you approach them, be relaxed and hide your intentions.
- Do not forget food.

www.soidog.org

Lifting and Carrying

- When lifting, a dog should always be supported at both ends. Larger dogs will require two, or more people to lift.
- The dog could be held by scruff of neck and supported underneath stomach. By "scruffing", dogs are less able to bite, beware scruffing brachycephalic type dogs.
- Muzzle the dog if you are not sure if it will bite.

Materials and Methods

- Slip leash
  - Used for only "friendly" dogs.
  - Used for catching and temporary restraint.
  - Can be used as a quick, temporary muzzle to prevent biting.
- Net and holder
  - Useful for small/medium sized dogs, not advisable for cat.
  - Will need more AROs to help in the wild area/ jungle.
  - All AROs must be able to use net and holder confidently.
  - Every pickup truck must have 2 net and holder in the truck.
  - Transfer dog from net and holder to cage gently and do not throw the animal with net in cage.
- Traps
  - Individual trap and multiple traps are humane method.
  - Ideal for shy animals or herd of feral dogs.

www.soidog.org

- Best method for cats.
- They can also be used for transporting animals.
- Traps should be placed away from where they can be seen by members of the public. Or there must be a public sign for prevent people intervene, and the use of labels also helps, explaining their purpose and who has set the trap.
- For individual trap, it is also better to place the trap against a building or large object and in the shade rather than out in the open.
- Be sure it will remain in good ventilation, not in the sunshine all day, and safe for animal.
- Talk to local residents or feeders to get permission if private property is a good location. They will often watch and protect the trap for you.
- Check the traps at least twice a day and list where every trap is located.

- Chemical capture
  - Should be used as last resort and not for cats.
  - DO NOT dart dogs who weigh less than 5kg, brachycephalic dogs and sick dogs.
  - The ARO will be able to adjust the dose of chemical depending on the condition of dog, reducing for dose for dogs that are slightly underweight or suspected to be ill (not fit to sterilise, but should be taken to BKK Clinic or PTS at Mobile if beyond help ; darting these types of dogs is the last resort).
  - Blow-pipe is most acceptable method of darting/ chemical capture in Thailand.
  - Follow the drug instruction for darting in **CNVR/SOP014.1**
