# Supplementary material for: Impact Assessment of Free-Roaming Dog Population Management by CNVR in Greater Bangkok
Source: Animals (Basel). 2023 May 23;13(11):1726. doi: 10.3390/ani13111726 (PMC10252118; doi:10.3390/ani13111726)
Supplement: Supplementary file 1 [file animals-13-01726-s001.zip › S2 - Street survey protocol.zip!/S2 - Street survey protocol.pdf]

<u>**Supplementary materials: Street survey protocol**</u>

Routes for surveying

In 2016, 20 routes were established across central Bangkok, these created a sample of 596km of streets that were unbiased with regards to free-roaming dog density (figure 1). A variety of road types were chosen in an attempt to generate a fair representation of streets, however elevated highways and major high speed multi-lane roads were excluded as these would be dangerous to survey. Main roads, smaller side streets (sois), 2-lane roads through urban residential areas, markets, temples and several rubbish dumps were included.

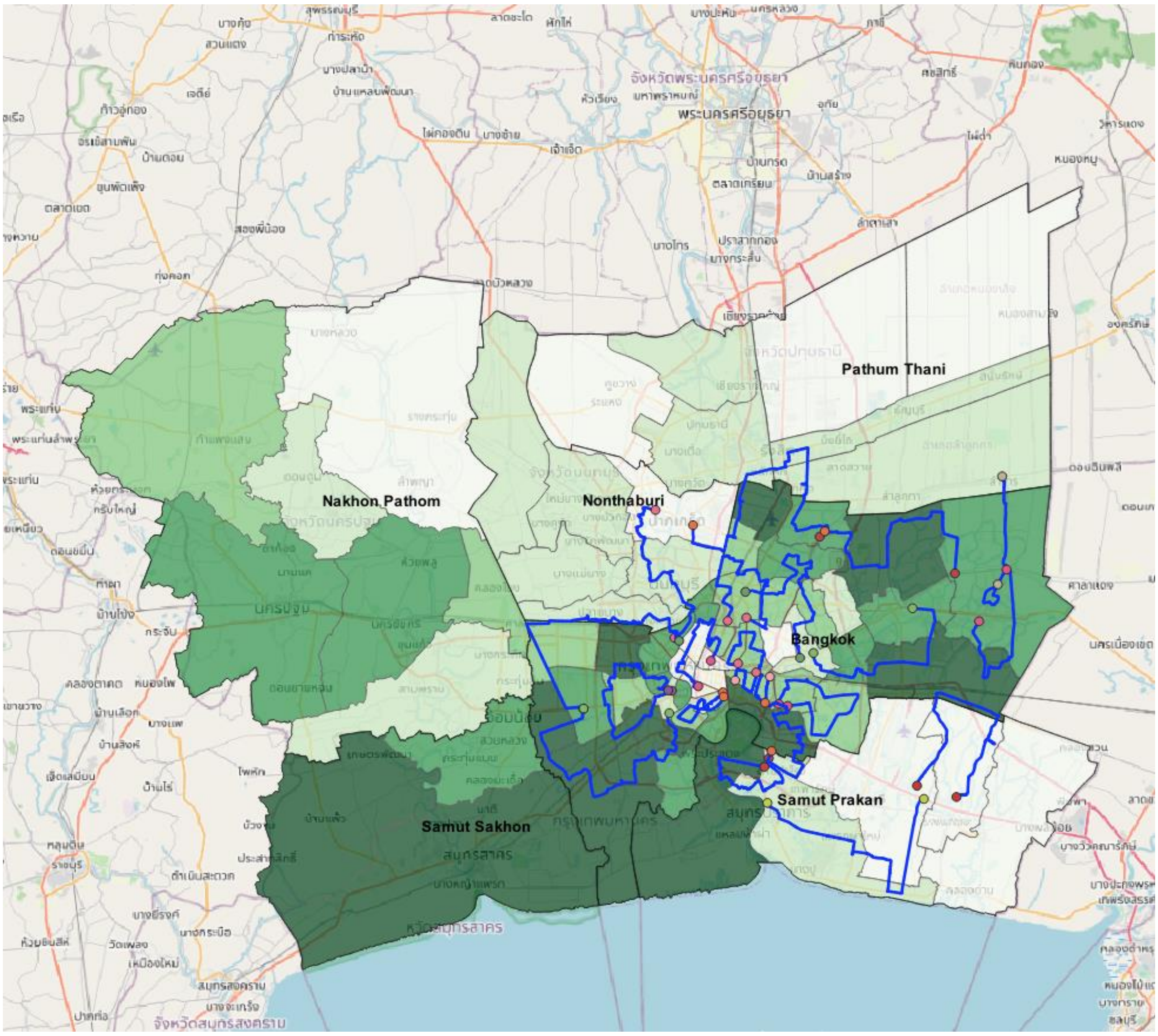

Figure 1. Map of Greater Bangkok showing the number of sterilizations per km of street in each of the districts; the 5 shades show increasing levels of sterilization effort per km of street, from 0 to 15.4 sterilizations per km in 5 equal steps. The names and boundaries of the Greater Bangkok provinces are overlaid in black. The 20 survey routes used for the street survey are overlaid in blue.

Prior to surveying in 2020, these routes were tested to ensure they were passable. There had been significant developments to the public transport system in Bangkok in the intervening years and some roads had changed. In addition, the surveyor in 2016 travelled by bicycle, whist the 2020 surveyors were on motorbike, this meant that some original route road crossings were not possible in 2020. Route detours and amendments were as follows:

- 6 routes could be followed without amendments or detours.

- 5 routes required the observer to follow part of the original route on foot whilst the motorbike driver completed a detour and then picked the observer back up as soon as possible.
- 9 routes require small amendments because the original route was impassable.

The total survey distance in 2016 was 596 km, the distance in 2020 was 613km, a 3% increase in distance. To compensate for the additional distance, analysis was conducted using dogs per km as the dependent variable rather than the number of dogs observed.

Observation protocol

Surveyors travelled along the survey route recording every free-roaming they observed on the street that made up the route, plus those dogs up side streets to a maximum distance of 20m. They moved as quickly as possible, avoiding interaction with dogs unless this was required to change their position in order to establish demographic or welfare attributes (see next section).

Dog demography and welfare attributes

For each dog observed, their GPS position was recorded along with the following 7 attributes. For each attribute there is an 'unknown' category for dogs who are only seen briefly or where their sitting/lying position obscures accurate judgement:

1. Sex
    - Male
    - Female
    - Lactating female (actively nursing puppies or obvious mammary tissue indicative of lactation)
    - Unknown sex
2. Age
    - Puppy (under 6 months of age or less than 6 kg)
    - Adult
    - Old (geriatric characteristics including saggy skin, grey muzzle, diminished general condition, stiff, wobbly)
    - Unknown age
3. Sterilisation
    - Sterilised (dog is recognised as having been through CNVR or there is a visible lack of testicles, tattoo in ear or shaved fur)
    - Intact (dog is recognised has having escaped CNVR efforts or there are visible testicles, pregnancy or breeding behaviours, such as females 'in heat')
    - Unknown sterilisation status
4. Body condition score using a 5-point score as outlined on ICAM website (www.icam-coalition.org/tool/dog-body-condition-scoring-training/).
    - BCS 1 – emaciated
    - BCS 2 – thin
    - BCS 3 – ideal
    - BCS 4 – fat
    - BCS 5 – obese
    - Unknown body condition
5. Skin condition
    - Healthy hair coat and skin
    - Mild to moderate hair loss or generalised dermal crusting
    - Severe skin disease
    - Unknown skin condition
6. Signs of ownership

   - Owned – Collar, t-shirt, human-dog interaction indicating ownership, including playing or being fed.
   - Unowned
   - Unknown ownership status
7. Signs of health
   - Healthy
   - Injured or ill – Included chronic wounds, TVT, neurological disease or lameness – anything that would require veterinary intervention under normal conditions.
   - Unknown health status

Consistency in protocol over time

To maximise robustness of results, the following aspects of the 2016 protocol were kept consistent in 2020 and 2021:

- Time of year – all surveys were completed in October and November.
- Time of day – all surveys started at 6am and finished before 10am, with one route completed by each survey team per day.
- Survey when weather is dry, postpone in case of rain.
- Speed was kept steady, pausing at each roaming dog to record its attributes.
- There was no attempt to attract dogs from other streets, only dogs on the route and up to 20m along side streets to the route were recorded.
- Routes were followed exactly, without unplanned detours (see 3.3 Route development for explanation of necessary planned detours in 2020).
- Dog type and welfare attributes were judged according to the same set of definitions developed in 2016.

The following protocol changes were made for 2020 and 2021:

- The 2016 survey was completed on bicycle, in 2020/21 the surveyors travelled by motorbike
- The 2016 survey was completed by one surveyor over 20 weekdays, in 2020 8 surveyors worked in pairs, creating 4 teams with the passenger principally responsible for observing and recording dogs whilst the driver navigated. On most days, more than one team was surveying simultaneously on different routes, hence the survey was completed over 10 days. In 2021, one surveyor took the lead role and completed every survey in partnership with one of 5 other surveyors.
- In 2016, the survey records were collected on pen and paper and then transcribed each evening into Excel. In 2020, the Talea app and web tool (developed by ICAM and Wise Monkey Foundation) was used to collect the data on mobile phones during surveying, with syncing of data to the Talea web on completion of each survey. Talea app was used because it supports accurate navigation along a predetermined route and robust recording and storage of the track travelled and dog survey data.
- Weekday surveying only, not weekends (people behave/travel differently at the weekends, effecting dog movement).
