# Supplementary material for: Impact Assessment of Free-Roaming Dog Population Management by CNVR in Greater Bangkok
Source: Animals (Basel). 2023 May 23;13(11):1726. doi: 10.3390/ani13111726 (PMC10252118; doi:10.3390/ani13111726)
Supplement: Supplementary file 1 [file animals-13-01726-s001.zip › S3 - Attitude survey in English.zip!/S3 - Attitude survey in English.pdf]

Preview / Test Mode

Back to Build | Preview Settings | Ignore Page Logic | Fire Actions | Record Response | View Comments | Desktop View

Attitudes Towards Street Dogs Questionnaire

Your answers to these questions, and those of other people we ask, will be used to help us understand how you and your community feel about the stray dogs that live around you. We won't ask for your name, so your answers will be anonymous. There are no right or wrong answers to these questions, so please answer as honestly as you can and you can skip any question that you do not wish to answer. The survey should take 10 minutes or less to complete.

1. What is your location? *

- ☐ Bangkok
- ☐ Samut Sakhon
- ☐ Nakhon Phatom
- ☐ Nonthaburi
- ☐ Samut Prakan
- ☐ Pathum Thani

2. What is your age group? *

- ☐ 10-16 years old
- ☐ 17-25 years old
- ☐ 26-35 years old
- ☐ 36-45 years old
- ☐ 46-60 years old
- ☐ > 60 years old

3. How much do you earn per month? *

- ☐ 0 - 7 500 baht
- ☐ 7 500 - 15 000 baht
- ☐ 15 000 - 25 000 baht
- ☐ 25 000 - 40 000 baht
- ☐ 40 000 baht or more

4. Do you own any dogs? *

- ☐ Yes
- ☐ No

5. Do you or anyone in your home currently feed any street dogs? *

- ☐ Yes
- ☐ No

6. In the last month, have you been annoyed or troubled by a dog or dogs in your neighbourhood? *

- ☑ Yes
- ☐ No

If Yes, what were the complaints? (more than one can apply) *

- ☐ Barking/Howling/Crying
- ☐ Pee and poo
- ☐ Make a mess of garbage
- ☐ Attacked my pet
- ☐ Biting me/ others
- ☐ Chasing me/ others
- ☐ Unsightly sexual behaviour
- ☐ Dog fights

- [ ] Chase me on my motorbike
- [ ] Destroy my property
- [ ] Saw a rabid dog
- [ ] Injured or sick dog
- [ ] Concern for puppies
- [ ] Other

7. How many dogs are roaming on your street today? *

- [ ] 0
- [ ] 1-3
- [ ] 4-6
- [ ] 7-10
- [ ] 11-20
- [ ] >20
- [ ] Don't know

8. What do you think about the number of dogs on your street? *

- [ ] Far too few
- [ ] Too few
- [ ] About right
- [ ] Too many
- [ ] Far too many
- [ ] Don't know

9. Thinking of the of dogs on your Soi today – and then thinking back 4 years ago to 2016 – which of the following statements is most true: *

- [ ] There used to be lots more dogs on my Soi
- [ ] There used to be far fewer dogs on my Soi
- [ ] The number is about the same
- [ ] I only moved here recently
- [ ] Don't know

10. Thinking of the of dogs on your Soi today – and then thinking back 4 years ago to 2016 – which of the following statements is most true: *

- [ ] I used to be more troubled by the dogs on my Soi
- [ ] I used to be less troubled by the dogs on my Soi
- [ ] I troubles I have with the dogs on my Soi are about the same
- [ ] I only moved here recently
- [ ] Don't know

11. What do you think should be done about roaming dogs? *

- [ ] Left alone
- [ ] Sterilisation
- [ ] Taken away to live in a shelter
- [ ] Rehomed as pets
- [ ] Other
- [ ] Don't know

12. Have you ever visited a dog shelter (in Thailand)? *

- [ ] Yes
- [ ] No

Next
